# A bibliometric and visual analysis of the use of ustekinumab in Crohn’s disease using CiteSpace

**DOI:** 10.3389/fphar.2023.1322319

**Published:** 2024-01-10

**Authors:** Yi Chen, Jiaqi Zhang, Junling Wu, Hanwen Zhang, Zhe Luan, Zhizhuang Zhao, Congyong Li, Yiming Zhao, Hu Zhang, Shufang Wang, Gang Sun

**Affiliations:** ^1^ Department of Gastroenterology and Hepatology, First Medical Center, Chinese PLA General Hospital, Beijing, China; ^2^ Department of Geriatric Medicine, Hainan Hospital, Chinese PLA General Hospital, Sanya, Hainan, China; ^3^ Department of Geriatric Gastroenterology, Sixth Medical Center, Chinese PLA General Hospital, Beijing, China; ^4^ Department of Gastroenterology and Hepatology, Hainan Hospital, Chinese PLA General Hospital, Sanya, Hainan, China; ^5^ China Medical, Sanofi, Beijing, China

**Keywords:** ustekinumab, Crohn’s disease, bibliometric analysis, biomarker, safety

## Abstract

**Background:** The novel biologic agent ustekinumab (UST), a monoclonal antibody against the p40 subunit of interleukin-12 and interleukin-23, has been applied in the treatment of Crohn’s disease (CD). With the development of relevant research, the clinical treatment and favorable prognosis of UST in CD have garnered considerable attention. However, there is a lack of reports that present the current status of UST-related studies in a comprehensive and objective manner. Consequently, this study aims to visually analyze the current status and clinical trends of UST-related research, identify leading researchers, and recognize deficiencies using bibliometrics and knowledge mapping, which might assist in understanding future research priorities in that specific field.

**Methods:** Published articles containing the use of UST in CD were retrieved from the Web of Science core collection database between 2008 and 2022. Then, the bibliometric analysis was performed, and a knowledge map was generated and visualized using the CiteSpace software.

**Results:** A total of 479 articles published between 2008 and 2022 were included in the bibliometric analysis. These publications were authored by 185 scholars from 51 countries or regions, among which the United States (38.3%), Canada (16.9%) and England (10.0%) were predominant in publishing. The keyword analysis indicated that UST has long been a popular biologic agent, and its clinical efficacy, safety, and indication for vulnerable populations in CD are popular research topics. The phrase “fecal calprotectin,” a biomarker reflecting the degree of disease activity and monitoring the therapeutic response, began to gain traction in 2020 and has continued to this day. Looking for UST-related biomarkers was gaining clinical attention.

**Conclusion:** The number of clinical studies involving the outcome of UST treatment in CD patients has increased, with the current research focusing on efficacy, safety, indications for vulnerable populations, therapeutic drug monitoring, and biomarkers. As an alternative drug after the failure of traditional immunosuppressive therapies or TNF-α antagonist therapy, UST is an effective and safe therapy in real-world refractory CD patients. UST will remain an active candidate for research in the treatment of CD.

## 1 Introduction

Crohn’s disease (CD), characterized by symptomatic progression in a relapsing-remitting manner, is a chronic non-specific inflammatory intestinal disease of unknown etiology, resulting in intestinal damage and debility (Torres et al., 2017). The incidence and prevalence of CD increase annually on a global scale, resulting in increased rates of hospitalization and disability that have a negative impact on the quality of life of patients.

Further, it brings disproportionately financial and medical burdens to the patient’s families and society (Kaplan, 2015). The general treatment primarily consists of 5-aminosalicylic acid preparations, steroids and immunomodulators (Cushing and Higgins, 2021). However, their therapeutic efficacy is not always satisfactory. With the in-depth research into pathological mechanisms of CD, dysregulated immune function, caused by cytokine-mediated inflammatory responses, is considered critical to intestinal inflammation. The main cytokines involved in inflammation are tumor necrosis factor-α (TNF-α), IL-12, and IL-23 (Geremia et al., 2014). Subclinical inflammation often persists even during clinical remission, increasing the risk of complications such as penetration or stricture (Jones et al., 2008). Therefore, the prevention of inflammatory responses has now become a therapeutic objective beyond symptomatic treatment. Based on this evidence, a wide range of biologics have been developed to treat CD. In addition to the classical TNF-α antagonists such as infliximab (IFX), and adalimumab (ADA), other biologics are frequently used in clinical practice, including anti-integrin agents (vedolizumab), and interleukin 12/23 antagonists (UST). The advent of biologics has made endoscopic and histological remission realistic for patients with CD.

Ustekinumab (UST) is a fully-humanized monoclonal antibody against the p40 subunit of interleukin-12 and interleukin-23. Under normal conditions, the cytokines IL-12 and IL-23 participate in intestinal immune homeostasis. When IL-12/23 is synthesized excessively as a result of the intestinal microflora imbalance, it accelerates the immune response in the intestine by activating innate immune cells, thereby evoking an early inflammation of CD (Greving and Towne, 2019). The inhibition of the IL-12/IL-23 pathway, triggered by UST, results in the alteration of T-cell differentiation, ultimately leading to reduced Th1 and Th17 pro-inflammatory cytokine production (Moschen et al., 2019). In 2009, the US Food and Drug Administration (FDA) authorized UST for the treatment of plaque psoriasis; in 2017, the clinical indications of UST were expanded into the treatment of moderate to severe active CD in naïve patients and also in those previously exposed to immunosuppressants and/or biologics. The use of UST was approved by the European Medicines Agency in the same year (Aggeletopoulou et al., 2018). In China, UST became available for CD patients when the National Medical Products Administration (NMPA) approved it in 2019. The 2021 AGA Clinical Practice Guidelines strongly recommended the use of UST in patients with moderate to severe CD for inducing and maintaining remission (Feuerstein et al., 2021). UST-related research is currently a trending topic due to its immense advantages in clinical therapy and favorable prognosis, especially in patients after the failure of TNF-α inhibitor therapy. Overall, a comprehensive analysis of UST-related studies will provide insights into its treatment progress in CD.

The bibliometric analysis uses statistical methods and visualization techniques to advance our knowledge structure on the progression of a specific field through qualitative and quantitative analysis of the scientific literature (Smith, 2008; Zhang et al., 2020). CiteSpace, a Java-based application, widely used in the field of information science, evaluates the distribution of publications by authors, institutions, countries, and journals to provide the research status of a topic and the leading researchers in that particular field (Chen, 2004). Over the past decades, numerous researchers and institutions have been committed to performing clinical research related to UST in CD. Based on the Web of Science core collection, this study thoroughly utilizes the bibliometric software program, CiteSpace, to visually construct a knowledge map to grasp the basic situation, research hotspots, and development trends in UST therapy in CD research and to lay a foundation for the direction of future research.

## 2 Methods

### 2.1 Data sources and search strategy

**FIGURE 1:**
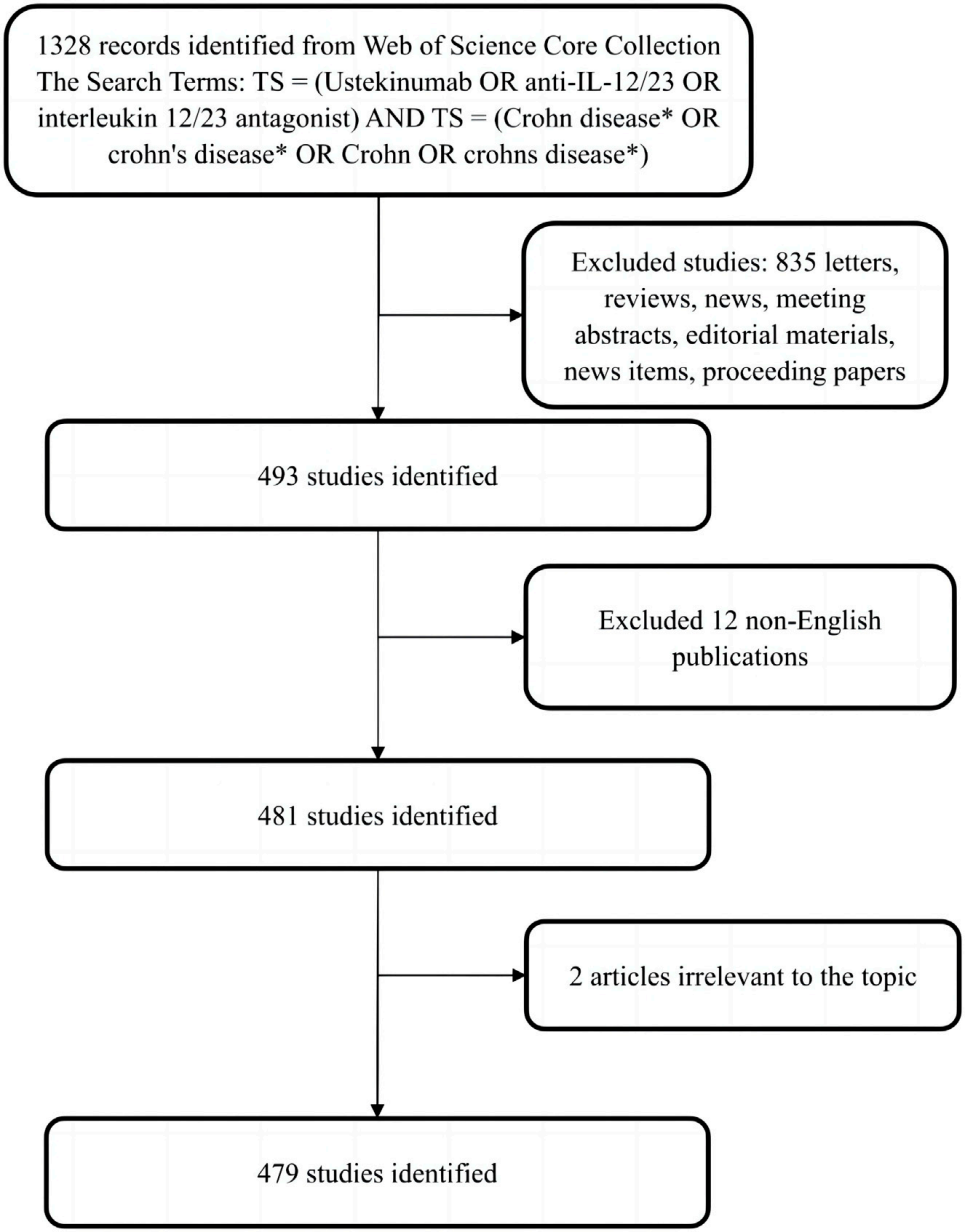
Flowchart of literature selection and the steps of bibliometric analysis included in this study.

Web of Science (WOS) is one of the most comprehensive and authoritative databases that provides bibliometric information on more than 12,000 high-quality journals from various countries, which can be used for analyzing and visualizing scientific literature (Wu et al., 2021). Data were extracted from the Web of Science core collection (WoSCC) and downloaded on 5 January 2023. The search formula was set to TS = (Ustekinumab OR anti- IL-12/23 OR interleukin 12/23 antagonist) AND TS = (Crohn disease* OR crohn’s disease* OR Crohn OR crohns disease*), and the date of the publications was from 1 January 2008, to 31 December 2022. A total of 1328 articles were retrieved, and a flow chart of the literature included in this study was provided (Figure 1). Only articles written in English were included. Other than full publications, such as conference abstracts and editorial materials, were excluded to ensure the accuracy and objectivity of the analysis. Eventually, 479 publications associated with the field were exported as “full records and references” into CiteSpace to analyze and visualize the scientific literature.

### 2.2 Analysis tool

All valid information retrieved from the WoSCC was input to Microsoft Excel 2021 and CiteSpace for visualization and summarizing. Microsoft Office Excel 2021 was employed to analyze the trends in the number of articles published. CiteSpace, a bibliometric citation visualization analysis software, developed by Professor Chaomei Chen of Drexel University using Java language based on bibliometrics, visual analytic methods, and data mining algorithms, helped to gain deep insights into a specific field (Zhu et al., 2020; Ma et al., 2021). Apart from summarizing the distribution of articles by countries/regions, authors and co-cited authors, journals and co-cited journals, co-cited references, keyword cluster analysis, and timelines, this interactive analytic tool also generated collaboration network analysis, co-citation analysis, and co-occurrence analysis in the form of visual knowledge maps (Ma et al., 2021; Chen, 2017). As a growing field of information technology, knowledge network maps offer research hotspots and evolution processes that are easy to interpret by researchers and forecast the developmental trends of different fields (Donnelly, 2017). CiteSpace has one of the most significant features called burst detection, which can be used to identify emerging trends and research frontiers by extracting burst words from titles, abstracts, and descriptors and identifiers of bibliographic records (Liu et al., 2019). In summary, CiteSpace can provide information on the current status, hotspot areas, leading researchers, and development trends in a specific field through a knowledge map.

## 3 Results

### 3.1 The trends of publication outputs

**FIGURE 2:**
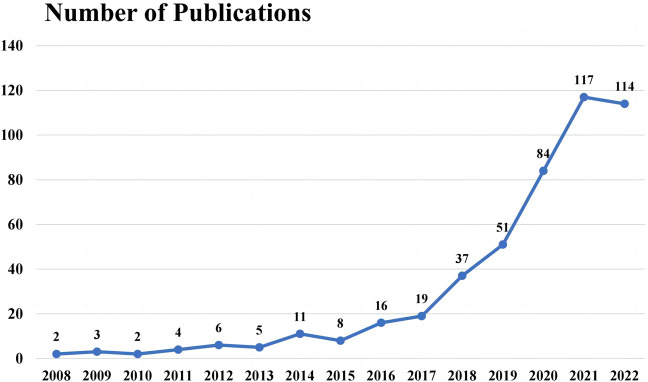
Change trend of annual number of publications related to the research on ustekinumab treatment in CD, 2008–2022.

The past 14 years, from 2008 to 2022, has witnessed a surge in annual publication numbers, with a total of 479 scientific articles published globally in science citation index (SCI) journals. As shown in Figure 2, the yearly publication concerning UST therapy within the CD research domain was overall on an upward trend, from 2 articles in 2008 to 114 articles in 2022, peaking at 117 articles in 2021. The cumulative annual publications suggest that the treatment of UST is a considerable research focus over the last decade, indicating it as a potential area of ongoing interest in the scientific community for the foreseeable future.

### 3.2 Distribution of countries/regions and institutions

A collaborative network map created by CiteSpace showed each node as a country/region, institution, or author whose radius increased with its contribution to UST-related research. Thicker lines indicated stronger co-occurrences or co-citations between nodes. The distinction between different years was depicted by various colors. In CiteSpace, “centrality” refers to intermediate centrality, which is an indicator of the importance of each network node. If the intermediate centrality is not less than 0.1, then that node is surrounded by a purple circle to show the high centrality, while a red circle shows the high emergence (Liu et al., 2019; Ma et al., 2021).

**FIGURE 3:**
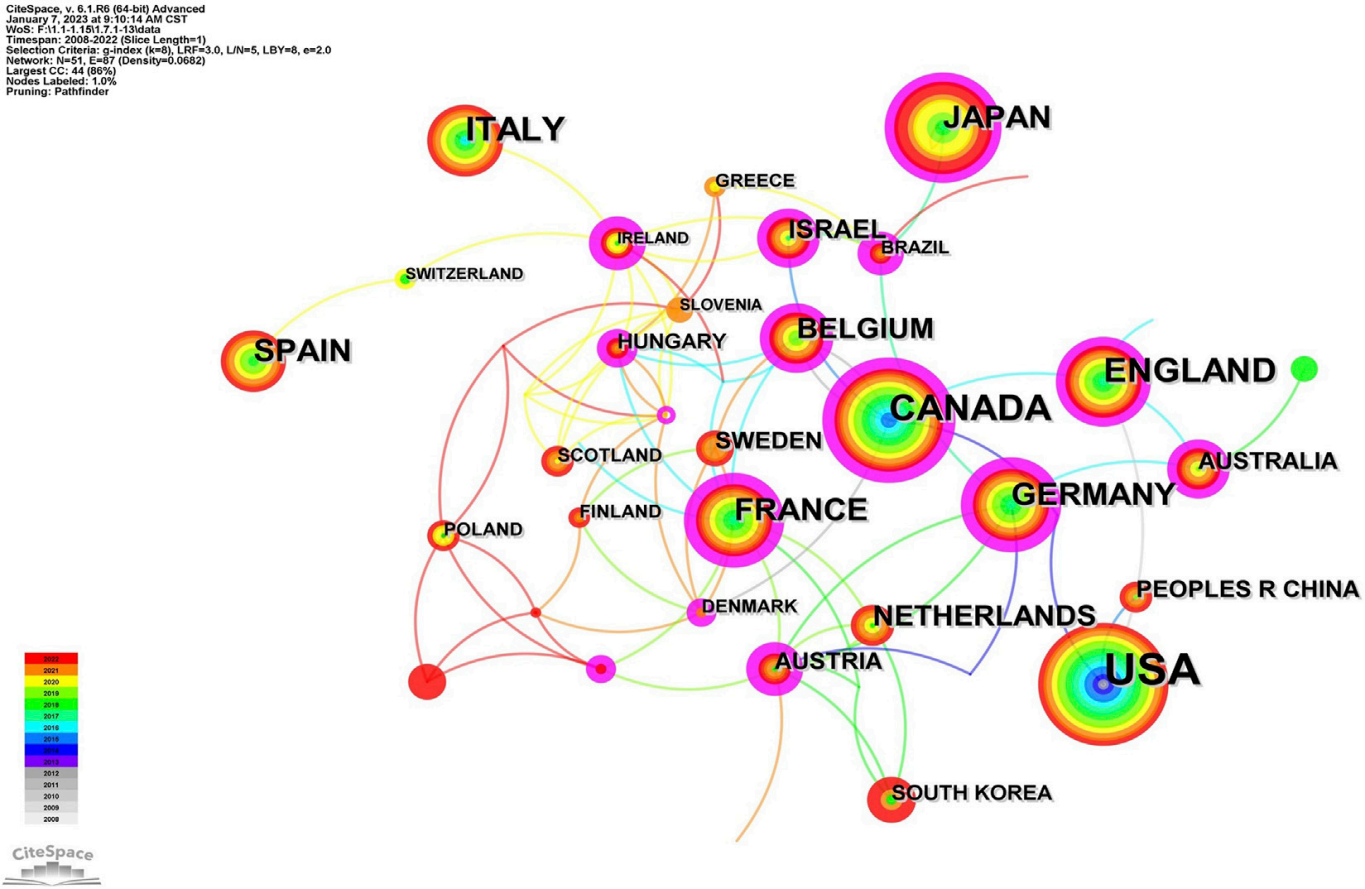
Map of a subset of cooperative relations among countries, 2008–2022.

In Figure 3, the collaborative network of countries/regions had 51 nodes and 87 connections with a density of 0.068251, meaning that 51 countries/regions were involved in elucidating the clinical application of UST in CD. As described in Figure 3 and Table 1, United States (*n* = 186) ranked first in the SCI research articles, followed by Canada (*n* = 81), England (*n* = 48), Italy (*n* = 47), and Japan (*n* = 45). On the whole, North American and European countries led the corresponding research and have close cooperation with each other. In contrast, Asian publication volume was slightly lower, dominated by Japan, Korea, and China. In addition, these three nations were less interconnected, indicating a lack of cooperation and communication.

**TABLE 1:** The top 10 prolific countries/regions and corresponding institutions.

Rank	Country	Year	Centrality	Count	Institution	Year	Centrality	Count
1	United States	2008	0.07	186	Icahn School of Medicine at Mount Sinai (United States)	2014	0.14	32
2	CANADA	2008	0.29	81	Western University (CANADA)	2018	0.33	31
3	ENGLAND	2010	0.18	48	University of California, San Diego (United States)	2012	0.09	29
4	ITALY	2010	0.07	47	Janssen Pharmaceuticals (United States)	2018	0.05	26
5	JAPAN	2017	0.13	45	Katholieke Universiteit Leuven (BELGIUM)	2019	0.03	23
6	FRANCE	2016	0.27	42	University of Calgary (CANADA)	2012	0.13	21
7	GERMANY	2011	0.25	40	University Hospital Leuven (BELGIUM)	2018	0.01	19
8	SPAIN	2008	0	35	Université Paris Cité (FRANCE)	2020	0	17
9	BELGIUM	2008	0.13	33	University OF Amsterdam (NEDERLAND)	2018	0.19	17
10	NETHERLANDS	2017	0.01	25	McMaster University (CANADA)	2019	0.02	16

**FIGURE 4:**
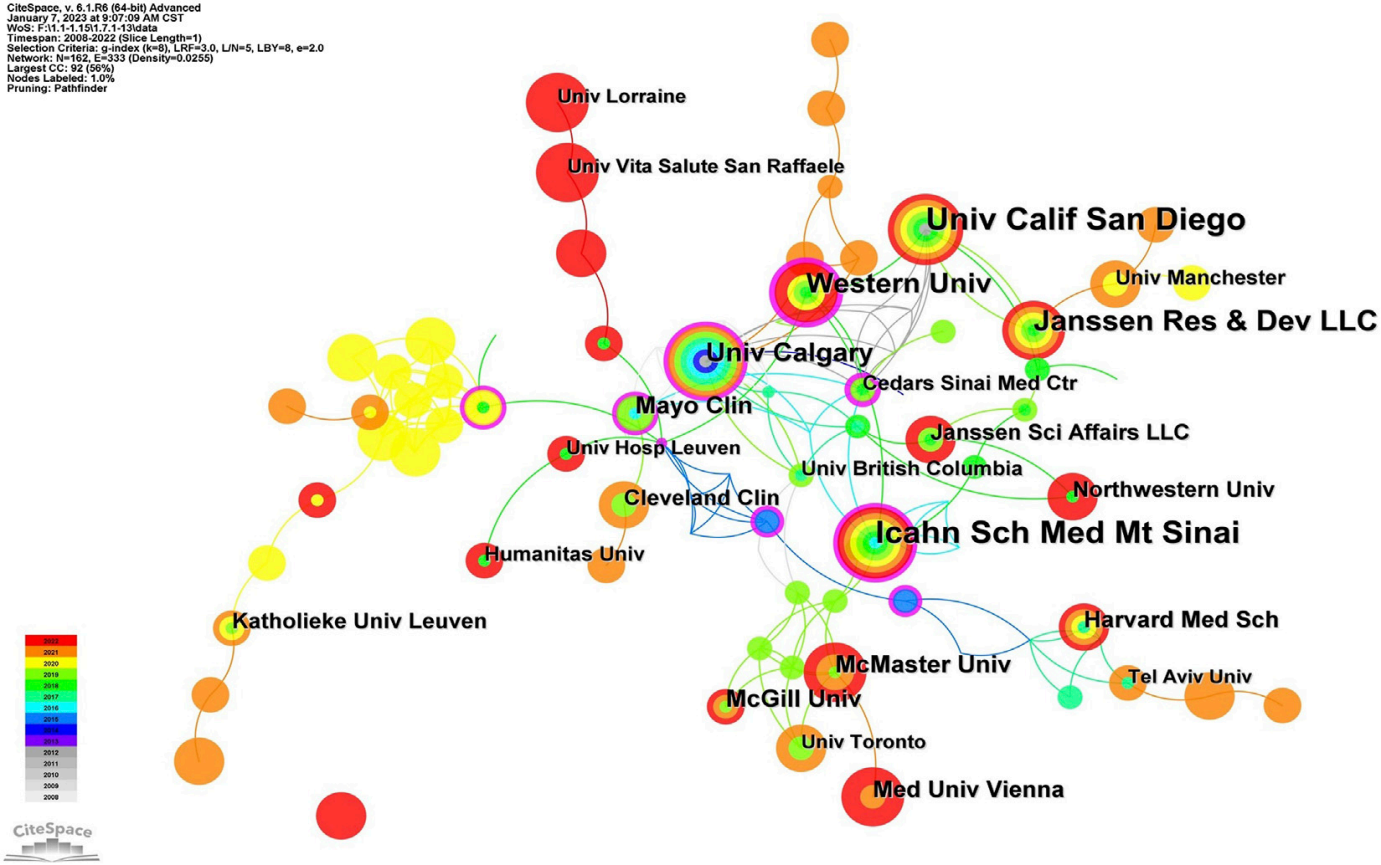
Map of a subset of cooperative relations among institutions, 2008–2022.

An analysis of institutions publishing articles and their collaborative relationships is necessary to acquire information on core institutions in a certain field of research. Figure 4 displays the information on co-institutes in the field of UST-related research. There were 162 institutions and 333 links between institutions on the knowledge map, with strong collaboration between institutions and significant contributions to the field from each country. According to Table 1, the Icahn School of Medicine at Mount Sinai was the most productive institution (*n* = 32), followed by Western University, Canada (*n* = 31), University of California-San Diego (*n* = 29), and Janssen Pharmaceuticals (*n* = 26). Besides, the Icahn School of Medicine at Mount Sinai, Western University, and the University of Calgary have greater centrality, demonstrating that they collaborate extensively with academic institutions. Geographically, most leading institutions were from the United States and Canada, further signifying the robustness of UST-related research in these countries. The reasons can be attributed to the following factors: The high prevalence of Crohn’s disease in North America drives the region’s researchers to explore and evaluate innovative therapies like UST. Besides, North America boasts advanced research infrastructure, and is home to a significant number of experienced gastroenterologists specializing in inflammatory bowel disease (IBD), such as William J. Sandborn and Brian G. Feagan. These countries and institutions worked together to carry out a series of randomized controlled trials, such as the UNITI series and the SEAVUE study, to assess the clinical efficacy and safety of UST and to generate more real-world follow-up data from the above studies.

### 3.3 Authors and co-cited authors

**FIGURE 5:**
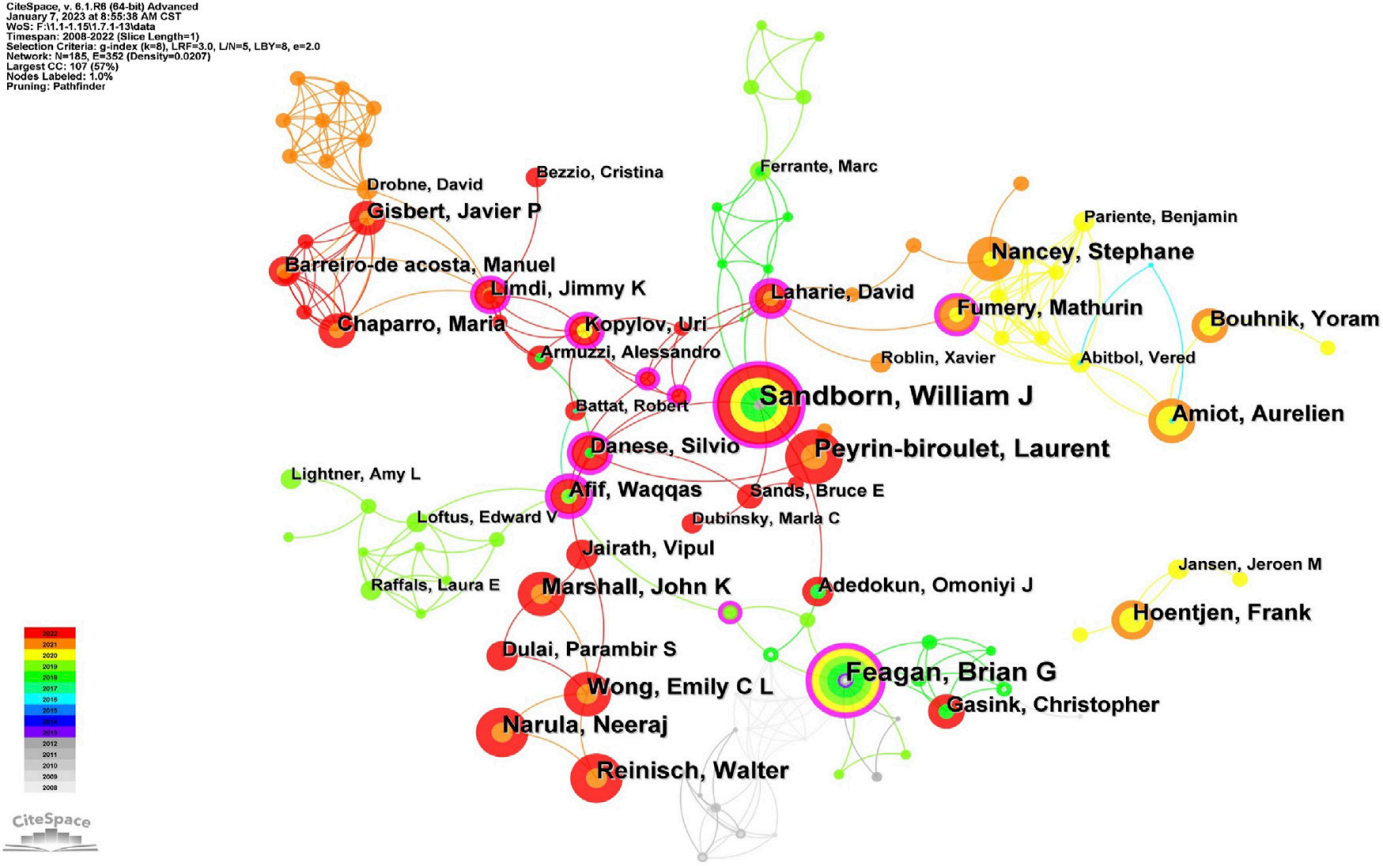
The network of authors, 2008–2022.

Co-cited authorship analysis pertains to the occurrence wherein a third author cites the works of two different authors in the same context (Cheng et al., 2021). The Highly Cited Index (H-index), first suggested by Jorge Hirsch, is a measure of a researcher’s scientific publications’ productivity and citation impact (Costas and Bordons, 2007). CiteSpace constructed a network graph mapping authors’ collaboration, and 185 authors published articles about UST-related clinical research in CD patients. (Figure 5). Table 2 and Table 3 shows the top 10 productive authors and co-cited authors in terms of number of publications, co-citations, centrality, H-index, and the corresponding countries. William J. Sandborn (University of California San Diego School of Medicine, United States) is the most productive and frequently co-cited author, who had the highest H-index with 25 papers. Graduating from Loma Linda University School of Medicine, William J. Sandborn is one of the world’s top experts in treating Crohn’s disease. He saw the potential of using these biologics in treating patients with inflammatory bowel disease (IBD) that lacked efficient treatment and innovatively designed multicenter clinical trials and evaluated the outcomes for most of the drugs widely used today.

**TABLE 2:** The top 10 most productive authors.

Rank	Author	Publications	Citations	H-index	Country
1	William J Sandborn	25	99,155	153	United States
2	Brian G Feagan	16	48,149	101	CANADA
3	Stephane Nancey	14	635	13	FRANCE
4	Remo Panaccione	13	31,341	76	CANADA
5	Walter Reinisch	13	24,964	68	AUSTRIA
6	Laurent Peyrin-Biroulet	13	4993	26	FRANCE
7	Christopher Gasink	13	2600	15	United States
8	Aurélien AMIOT	12	4267	33	FRANCE
9	Mathurin Fumery	11	3626	32	FRANCE
10	Subrata Ghosh	11	20,453	63	IRELAND

**TABLE 3:** The top 10 co-cited authors.

Rank	Co-cited author	Co-citations	Centrality	H-index	Country
1	William J Sandborn	274	0.2	153	United States
2	Brian G Feagan	255	0.03	101	Canada
3	Bruce E Sands	146	0.09	27	United States
4	Jean-Frédéric Colombel	118	0.37	113	United States
5	Stephen B Hanauer	118	1.14	2	United States
6	Kopylov Uri	87	0.64	46	ISRAEL
7	Christopher Ma	85	0	26	United States
8	Pauline Wils	75	0.34	24	ISITE
9	Robert Battat	74	0.24	19	United States
10	Paul Rutgeerts	73	0.11	147	BELGIUM

The collaborations of the authors in UST-related studies are shown in CiteSpace (Figure 5), which provides a holistic view of the academic community. William J. Sandborn, Bruce E. Sands, Brian G. Feagan and other core authors worked collaboratively, and strong collaboration among these scholars were important for the comprehensive investigation of the field. In the domestic context, clinical research concerning UST treatment is primarily spearheaded by Qian CAO from Sir Run Run Shaw Hospital (Zhejiang University) and Jun YU from Li Ka Shing Institute of Health Science (the Chinese University of Hong Kong). Their focus centers on investigating the etiological mechanisms of CD and assessing the real-world clinical efficacy and safety profile of UST within the national clinical setting (Wang et al., 2016; Yao et al., 2021; Cao et al., 2022).

### 3.4 Co-cited journals and references

The journal’s prominence is indicated by its impact factor (IF) and Journal Citation Reports (JCR) quartile. The IF, which was first introduced by Eugene Garfield, founder of the Institute for Scientific Information, is frequently employed as a scientometric of academic journals (Garfield, 2006). A total of 342 journals published 479 articles on UST, and many journals were CD-related specialized periodicals. Table 4 summarizes the top 10 journals and co-cited journals in the number of articles, frequency of co-citation, corresponding IF (JCR 2022), and JCR quartile. About 50% of the journals and 90% of the co-cited journals came under the Q1 ranking. *Inflammatory Bowel Disease* (39 articles; IF-2022, 7.29; Rank, Q1) had the highest number of output publications, while *the New England Journal of Medicine* (371 citations; IF-2022, 176.082; Rank, Q1) was the most cited journal among them. The majority of UST-related articles were cited by influential international journals, indicating their significance worldwide. The higher the citation frequency of a publication, the more influential the journal is in the relevant research field. The journal *Inflammatory Bowel Disease* had the highest number of publication outputs and the third highest number of citations, indicating its significant contribution to the clinical investigation of UST in CD.

**TABLE 4:** The top 10 journals and co-cited journals.

Rank	Journal	Output	IF	JCR	Co-cited journal	Co-citations	IF	JCR
1	Inflammatory Bowel Disease	39	7.29	Q1	The New England Journal of Medicine	371	176.082	Q1
2	Journal of Crohns & Colitis	31	10.02	Q1	Gastroenterology	356	33.883	Q1
3	Alimentary Pharmacology Therapeutics	23	9.524	Q1	Inflammatory Bowel Disease	340	7.29	Q1
4	Clinical Gastroenterology and Hepatology	15	13.576	Q1	Journal of Crohns & Colitis	324	10.02	Q1
5	Digestive Diseases and sciences	14	3.487	Q3	Alimentary Pharmacology & Therapeutics	292	9.524	Q1
6	BMC Gastroenterology	11	2.848	Q4	The Lancet	272	202.731	Q1
7	Gastroenterology	9	33.883	Q1	American Journal of Gastroenterology	266	12.045	Q1
8	Journal of Clinical Medicine	9	4.694	Q2	GUT	255	31.795	Q1
9	Scandinavian Journal of Gastroenterology	9	3.027	Q4	Clinical Gastroenterology and Hepatology	249	13.576	Q1
10	Digestive and Liver Disease	8	5.165	Q2	World Journal of Gastroenterology	100	5.374	Q2

Academic success depended on highly cited articles, and the bibliometric analysis identified articles that had a crucial impact on the academic community (Brandt et al., 2019). Co-citation analysis assessed the progress of the scientific field and recognized the frontiers in the research area. Table 5 displays the top ten co-cited articles pertinent to the topic of this study. The paper titled “Ustekinumab as Induction and Maintenance Therapy for Crohn’s Disease” (Feagan et al., 2016) was published in *the New England Journal of Medicine* in 2016 as the outcome of the UNITI series research, ranking first with a citation count of 928. The main focus of the top 10 co-cited publications was on the efficacy and safety of UST in CD, its drug trough concentrations, pharmacokinetic profiles, clinical treatment goals with endoscopic remission, and histologic remission.

**TABLE 5:** Top 10 co-cited references related to ustekinumab.

Title	Journal	First-author	Publication year	Total citations
Ustekinumab as Induction and Maintenance Therapy for Crohn’s Disease	The New England Journal of Medicine	Brian G Feagan	2016	928
Ustekinumab Induction and Maintenance Therapy in Refractory Crohn’s Disease	The New England Journal of Medicine	William J Sandborn	2012	768
Association Between Ustekinumab Trough Concentrations and Clinical, Biomarker, and Endoscopic Outcomes in Patients With Crohn’s Disease	Clinical Gastroenterology and Hepatology	Robert Battat	2017	136
Pharmacokinetics and Exposure Response Relationships of Ustekinumab in Patients With Crohn’s Disease	Gastroenterology	Omoniyi J Adedokun	2018	124
Subcutaneous Ustekinumab Provides Clinical Benefit for Two-Thirds of Patients With Crohn’s Disease Refractory to Anti-Tumor Necrosis Factor Agents	Clinical Gastroenterology and Hepatology	Pauline Wils	2016	128
Ustekinumab for the Treatment of Refractory Crohn’s Disease: The Spanish Experience in a Large Multicentre Open-label Cohort	Inflammatory Bowel Disease	Sam Khorrami	2016	113
Clinical, endoscopic and radiographic outcomes with ustekinumab in medically-refractory Crohn’s disease: real world experience from a multicentre cohort	Alimentary Pharmacology & Therapeutics	Ma C	2017	108
IM-UNITI: Three-year Efficacy, Safety, and Immunogenicity of Ustekinumab Treatment of Crohn’s Disease	Journal of Crohns & Colitis	Stephen B Hanauer	2020	89
Long-term efficacy and safety of ustekinumab for Crohn’s disease through the second year of therapy	Alimentary Pharmacology & Therapeutics	William J Sandborn	2018	83
Ustekinumab for Crohn’s Disease: Results of the ICC Registry, a Nationwide Prospective Observational Cohort Study	Journal of Crohns & Colitis	Vince B C Biemans	2020	80

### 3.5 The analysis of hotspots and the frontiers

#### 3.5.1 Analysis of keywords

**FIGURE 6:**
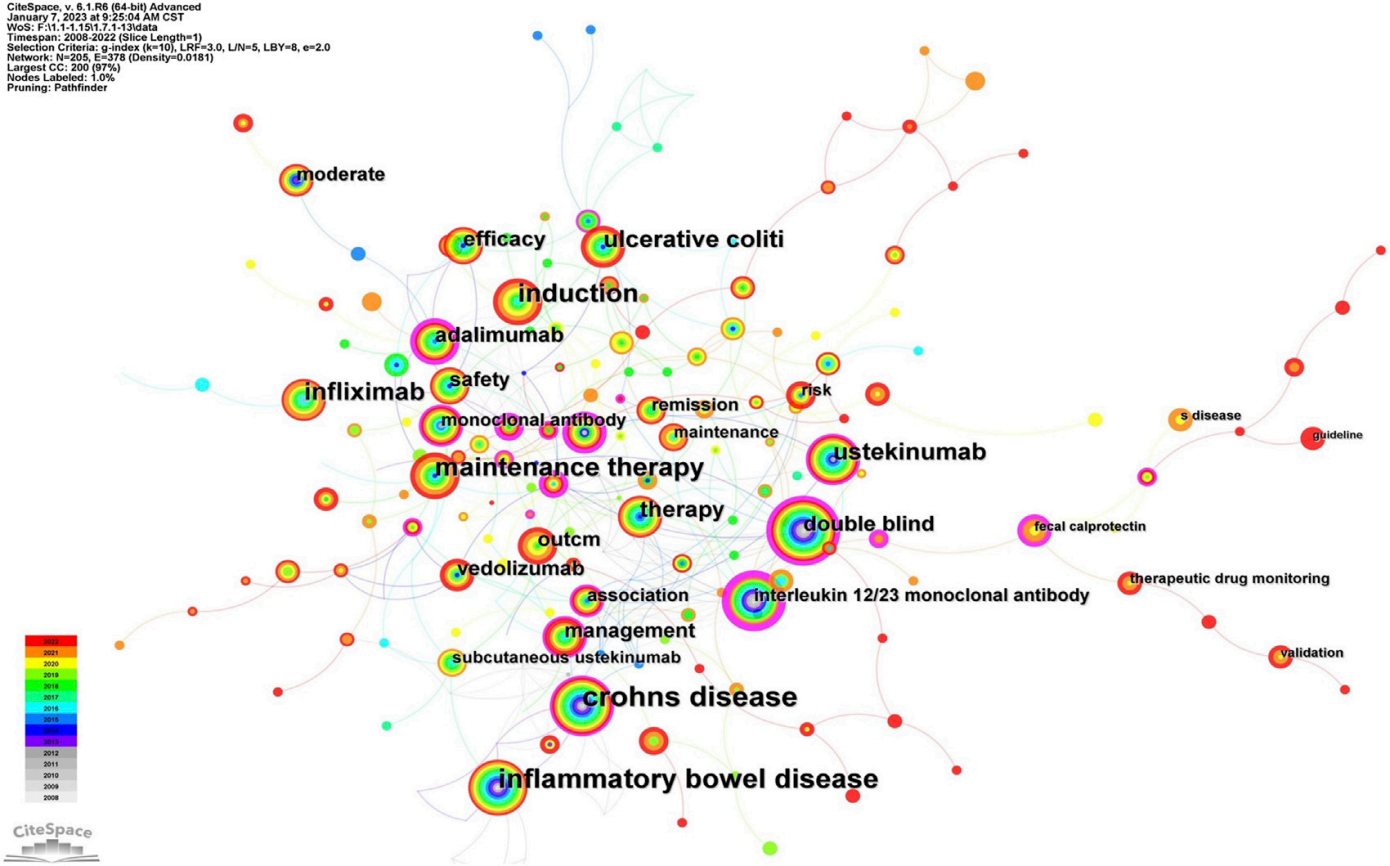
The keyword visualization map.

**FIGURE 7:**
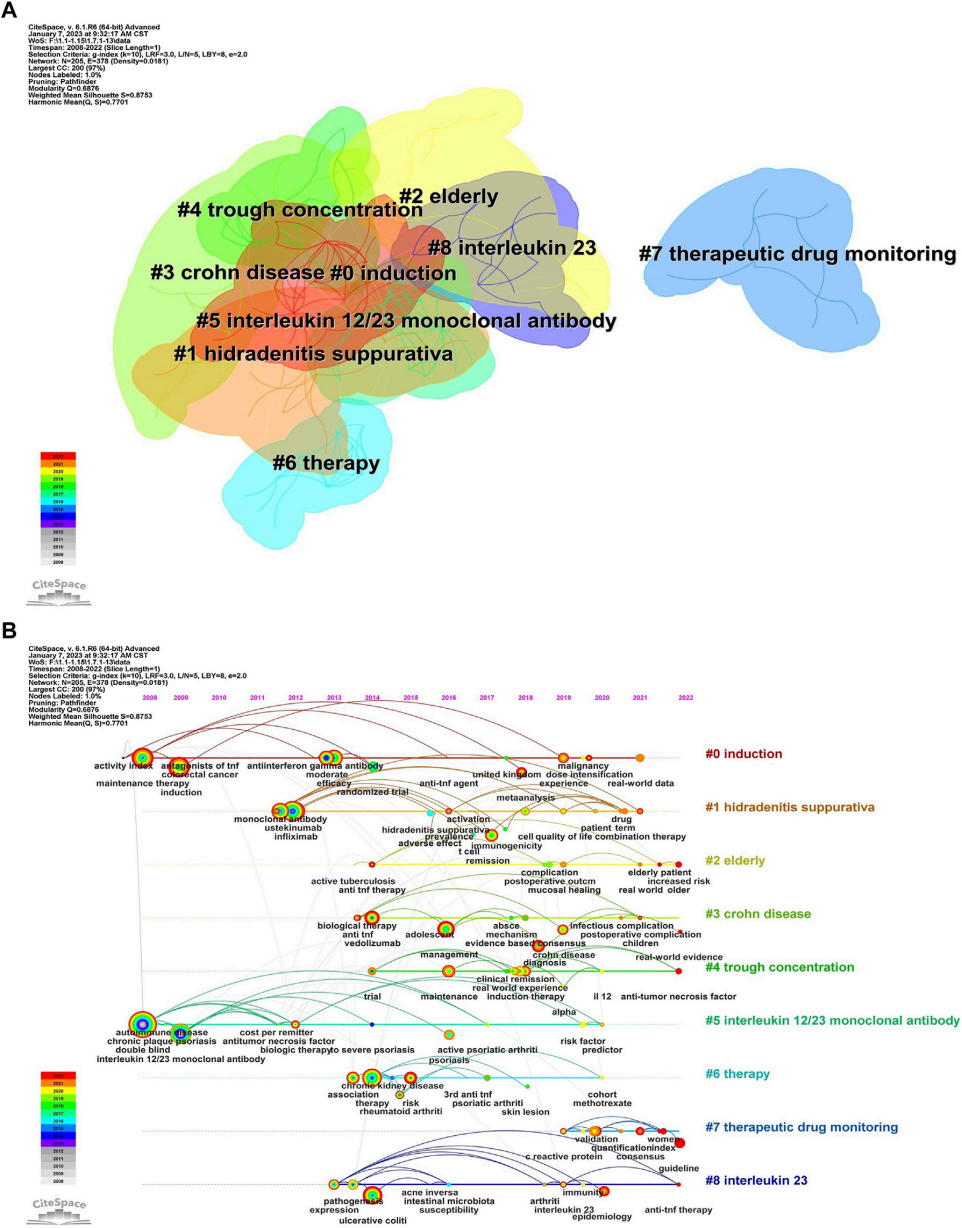
**(A)**. The cluster view map of keyword. **(B)**. The cluster timeline view map of keywords analysis.

**FIGURE 8:**
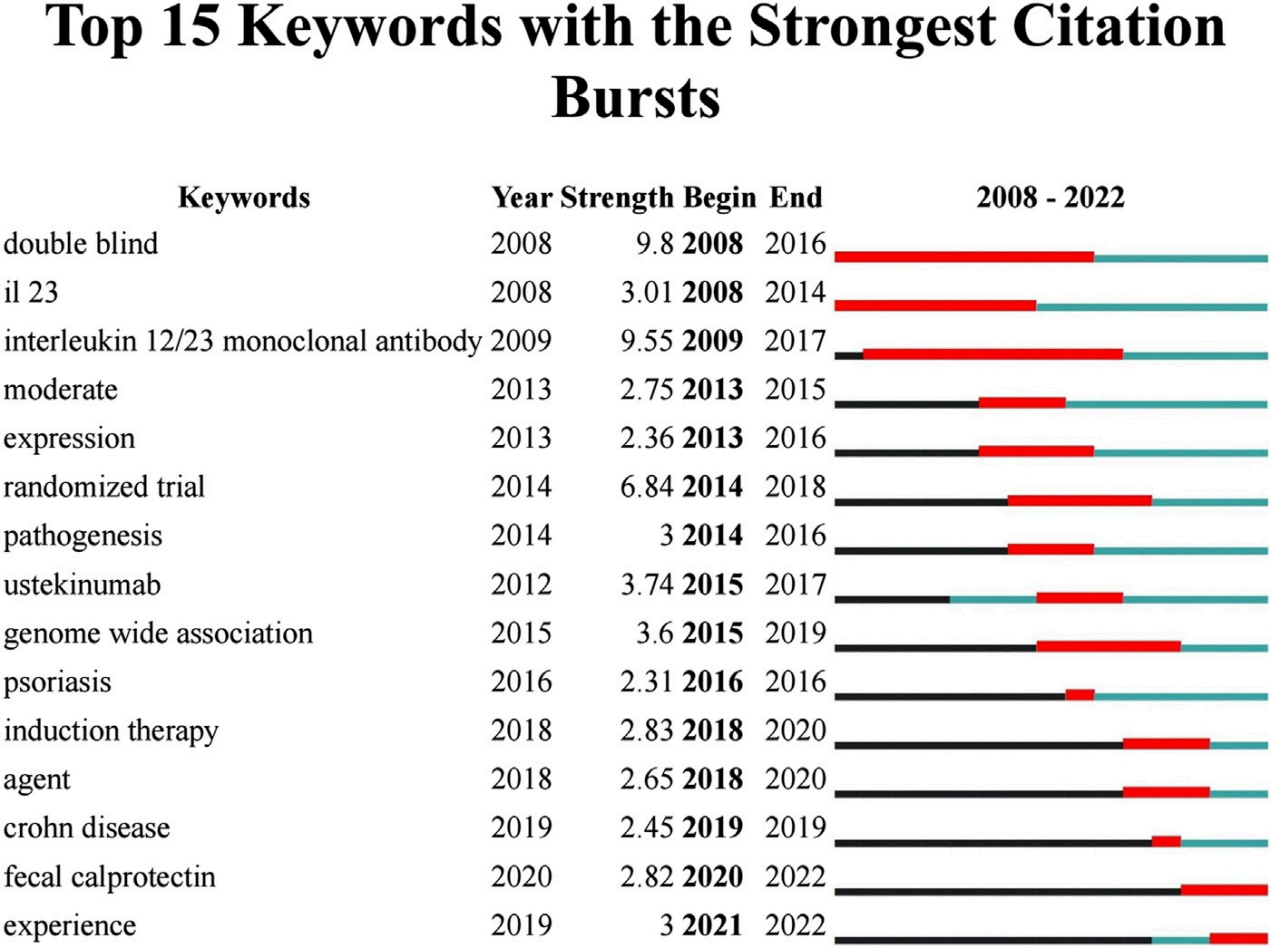
The top 15 keywords with the strongest citation burst.

Keywords, an overview of the core content of the article, can reflect the academic thoughts, topics, and contents of specific research. Through the analysis of keywords, the research hotspots and directions in the field were elucidated. CiteSpace presented a co-occurrence network diagram of keywords in two formats: Cluster View and Timeline View (Figure 6; Figures 7A, 8B). Combining the co-occurrence mapping of the keywords and the table of high-frequency keywords (Table 6), the keywords with high frequency in this field were “crohns disease” (276), “inflammatory bowel disease” (220), “maintenance therapy” (184), “induction” (151), “infliximab” (119), and “ustekinumab” (103). The clustering map of keywords provided an overview of the research interests in the field, while the timeline view of the clustering graph displays the most frequent keywords for each cluster over time based on the interaction and mutation relationship between them. As shown in Figure 7A, the keywords were clustered and roughly divided into 9 categories, numbered from 0 to 8, with most clusters overlapping. In Figure 7B, the timeline view is structured with the *X*-axis representing the publication year, and the *Y*-axis corresponding to the cluster number. It visually reflects the phased hotspots and developmental path of UST in CD research from the time dimension. Cluster 0 was the largest and contained the most items (28) which mainly related to the efficacy of UST in induction and maintenance therapy. One of the keywords is “dose intensification”, which is the focus of recent research. UST-treated patients with CD, who might exhibit insufficient response, or loss of response (LOR) following standard induction and/or maintenance dosing, can benefit from dose intensification. It is important to note that 5 of the 9 clusters, namely, #2, #3, #4, #7, and #8, are still concerned by researchers. Only recently attention has been paid to the clinical efficacy and safety of UST in vulnerable patients with CD, therapeutic drug monitoring (TDM) and biomarkers.

**TABLE 6:** The top 10 keywords.

Ranking	Keywords	Count	Centrality	Year
1	crohns disease	276	0.16	2009
2	inflammatory bowel disease	220	0.07	2009
3	maintenance therapy	184	0.08	2008
4	induction	151	0.02	2009
5	infliximab	119	0.04	2012
6	Ustekinumab	103	0.16	2012
7	ulcerative colitis	92	0.04	2014
8	therapy	72	0.06	2014
9	adalimumab	65	0.28	2009
10	double blind	61	0.38	2008

#### 3.5.2 Burst detection

The burst detection algorithm proposed by Kleinberg (Kleinberg, 2003) was the core function of CiteSpace to capture the turning points of keywords or references’ popularity within a specific period. The blue line represented the time interval, and the red line depicted the duration of the burst (Figure 8). The keywords with the highest burst intensity were “double-blind (9.8),” “interleukin 12/23 monoclonal antibody (9.55),” and “randomized trial (6.5),” dictating that these keywords were concepts of great significance to researchers in the period they corresponded to. “Fecal calprotectin,” an antimicrobial protein primarily secreted by neutrophils, started to explode in 2020 and remained bursting, and it is emerging as a suitable laboratory marker for the diagnosis and non-invasive management of IBD over traditional inflammatory biomarkers such as c-reactive protein (CRP) and erythrocyte sedimentation rate (ESR) (von Roon et al., 2007; Langhorst et al., 2008; Moein et al., 2017). Meanwhile, a growing number of studies confirmed that FC correlated well with major endoscopic evaluation scores and histopathological assessment (D’Haens et al., 2012). Treatment with UST improved FC levels in patients with CD (Ollech et al., 2021). An analysis of the correlation between FC level and endoscopic response in a prospective cohort trial revealed that a reduction in FC level (≥500 ug/g) at week 8 was associated with endoscopic response at week 16, which may guide therapeutic decisions on UST therapy in CD patients, including dose intensification or treatment discontinuation (Pauwels et al., 2023). However, more data on the extent to which FC levels can predict endoscopic response or histologic induction of remission in CD patients subjected to UST are still needed.

## 4 Discussion

### 4.1 General information

The WoS database was used in this study to conduct a comprehensive literature search for studies on UST treatment in CD that were published from 2008 to 2022.

The analysis in this study is based on 479 UST-related articles from 162 institutions with 165 authors in the WOSCC database from 1 January 2008, to 5 January 2023. The ongoing escalation in the total volume of published research articles indicates that UST is receiving a growing amount of attention. William J. Sandborn et al. first published the article “A randomized trial of ustekinumab, a human interleukin-12/23 monoclonal antibody, in patients with moderate-to-severe Crohn’s disease” in 2008, which began UST-related research. Over the past 10 years, there have been an increasing number of studies on UST therapy.

On a visual analysis of countries and institutions, the United States and Canada mainly contributed to this field. The United States ranked first in the number of published articles, followed by Canada and the United Kingdom. Among the top 10 countries (Table 1), Canada had the highest centrality (0.29), implying that it was a key bridge on the collaborative network map among countries. Of the top ten research institutions, three were from the United States, three were from Canada, and two were from Belgium, with Western University-Canada (0.33) having the highest centrality. Asian countries were dominated by Japan, South Korea, and China. Additionally, only a few nations participated in the research on the use of UST in CD globally, and collaboration and information exchange across groups were restricted to a small number of nations, which could impede the advancement of this field of study. Therefore, collaboration with institutions from other countries should be encouraged in developing UST for the treatment of CD to provide more real-world data from diverse populations, including African Americans, Latinx, Asians, and others.

From the perspective of authors and co-cited authors, William J. Sandborn (25 articles), from the University of California San Diego School of Medicine, United States, was the most published author and his co-citation frequency ranked first, which indicates his outstanding influence in UST-related fields. Brian G. Feagan was second only to William J. Sandborn in the number of publications and co-citations. He and William J. Sandborn first conducted a large randomized controlled trial (UNITI study) of IBD, and the outcome demonstrated the significant efficacy of UST in inducing and maintaining clinical remission in patients with moderate to severely active CD. Notably, the top 10 active and co-cited authors were almost all from the United States and Canada, signifying that North American researchers were at the forefront in the field of CD and contributed tremendously to the real-world evidence of UST therapy.

Publications in academic journals are often important achievements of scientific research, and the analysis of the source distribution of journals provides information for researchers to recognize the core journals in this field. According to the journals and co-cited journals (Table 4), *Inflammatory Bowel Disease* was the journal with the highest number of publications (39 publications), while *the New England Journal of Medicine* (371 co-citations) was the most cited journal. Four journals had more than 300 citations, and 50% of the journals and 90% of the co-cited journals came under the Q1 ranking. The cited journals tended to be the high-impact ones, indicating that research related to UST was highly valued from a global perspective.

### 4.2 Hotspots and frontiers

Keywords indicated the research theme and hot content of UST-related research. The keyword co-occurrence analysis recognized the research hotspot in a certain field. In addition to “ustekinumab” and “Crohn’s disease,” the representative words in Table 6 also included “maintenance therapy” and “induction,” which are the key stages with distinct treatment goals and strategies. The timeline diagram analysis shows that the hotspots of UST clinical research shifted over time. Efficacy, safety, TDM, indications for special populations, and biomarkers received more attention.

#### 4.2.1 Efficacy and safety

UST has supplanted anti-TNF therapy as the preferred first line biologic for CD for its comparatively favorable safety profile in a retrospective observational cohort analysis of all patients with IBD, according to a report from the European Crohn’s and Colitis Organization (ECCO) conference (FERNANDO et al., 2023). Based on the recent SEAVUE trial and network meta-analysis, UST treatment demonstrated similar efficacy in patients with moderate to severe CD compared with anti-TNF therapy (Sands et al., 2022). Additionally, UST may exhibit greater effectiveness than VED in patients who have previously failed TNF-α antagonists (Singh et al., 2021).

#### 4.2.2 UST LOR

A systematic review estimated the annual risk of UST LOR and the overall annual risk of LOR with UST among primary responders with CD to be 21% per person-year (Yang et al., 2022). The common cause of LOR to UST includes low UST trough concentrations and high anti-ustekinumab antibody concentrations. The low UST trough concentration and poor response to the UST treatment may be caused by variables including inadequate response to previous anti-TNF agents, wide-ranging intestinal lesions, profound ulceration, severe malnutrition, and high CRP levels (Verstockt et al., 2019). TDM can be useful in detecting patients with quick medication clearance and low trough concentrations in patients with an unsatisfactory clinical response or breakthrough symptoms. Both reinduction and interval reduction strategies have shown effectiveness in UST-treated patients with CD, who might exhibit insufficient response, or loss of response (LOR). The MUST (meta-optimization of Ustekinumab) study shows that severe CD patients who received prompt UST treatment dose escalation to 4-weekly dosing experienced better rates of clinical remission at 1 year than those who received 8-weekly medication Abdelmoula et al., 2023). The POWER study compared the efficacy and safety of a single intravenous (IV) re-induction UST dose vs. continued UST subcutaneous (SC) treatment in CD patients with LOR during UST maintenance therapy and revealed that a higher percentage of patients in the IV arm achieved normalization of FC, endoscopic remission, and improvement in the IBDQ score compared to the SC arm (Schreiber et al., 2023).

#### 4.2.3 Vulnerable population

Despite a scarcity of data regarding the efficacy of UST in clinical practice, vulnerable populations, including the elderly, pediatric patients, and pregnant women, are often underrepresented in clinical trials. Available studies have shown that UST is efficacious and safe in elderly patients and pediatric patients. In elderly patients, preference should be given to immunomodulatory therapies, namely, vedolizumab (VED) or UST, which exhibit a lower propensity for overall infection risk or malignancy (Ananthakrishnan et al., 2021). An observational and multi-center study suggests that UST appears to be equally effective in elderly patients with CD compared to non-elderly patients, and the safety profile is generally similar, except for a slightly higher incidence of *de novo* neoplasms, which can be attributed to the advanced age of the elderly patients (Casas-Deza et al., 2023). The existing data suggests that UST was effective and safe among pediatric CD patients at a single IBD referral center, with higher rates of remission observed in bio-naive patients compared to bio-exposed patients (Dayan et al., 2019). According to a retrospective multicenter study involving 25 sites affiliated with the IBD Interest and Porto groups of ESPGHAN, two-thirds of children with active CD responded to UST dose escalation (Yerushalmy-Feler et al., 2022). In addition, pregnant patients with CD who received UST demonstrated successful pregnancy and neonatal results that were comparable to those seen in patients receiving anti-TNF medications or other treatments (Avni-Biron et al., 2022). Nevertheless, the clinical efficacy and safety of UST therapy in vulnerable populations still lack real-world evidence and more data are needed to confirm them.

#### 4.2.4 Biomarkers

Biomarkers in the management and treatment of IBD have been the subject of extensive research over the past decade. There are different kinds of biomarkers on UST therapy in CD patients, like prognostic biomarkers, predictive biomarkers or biomarkers that predict the adverse events. CRP and FC are biomarkers of mucosal inflammation that are frequently used to evaluate disease activity in IBD. Several studies found an association between elevated CRP levels and an increased need for future surgery and a poorer long-term prognosis, including an increase in hospitalizations and intestinal resections (Henriksen et al., 2008; Oh et al., 2017). However, not all patients with active CD will exhibit elevated CRP levels, which is the reason of the growing utilization of faecal markers of inflammation, known for their higher sensitivity and specificity. FC is increasingly being utilized in the evaluation of diseases and recognized as monitoring tools for achieving target to treatment (Mosli et al., 2015). The effectiveness of FC in the prediction of endoscopic and histologic activity of IBD, and disease recurrence has been the subject of numerous investigations (Khaki-Khatibi et al., 2020). UST showed significantly higher FC remission rates compared to conventional IFX (53.8% vs. 19.2%, *p* = 0.020), and there was a trend towards lower hospitalization rates (7.7% vs. 30.8%, *p* = 0.075) in a propensity score analysis comparing UST to Infliximab and vedolizumab in CD from the ECCO conference (Matos Coelho Bernardo et al., 2023). However, available data on the degree to which FC levels can be used to predict the endoscopic response or histologic remission in UST-treated patients still lacks. In addition to the decrease in FC, the baseline serum levels of IL-23, IL-1, and IL-6 demonstrated high reliability in predicting the therapeutic outcome of UST for MH in CD. Due to technological advances, more and more types of biomarker, including genetic mutation, genetic mutations and microbiome can be identified by researchers (Verstockt et al., 2022). These biomarkers could help clinicians improve the management of UST therapy by identifying patients with a higher likelihood of responding to treatment earlier.

### 4.3 Research gaps and recommendations for future work

Even though UST-related clinical studies gained huge popularity, some gaps in the literature were identified. Clinical efficacy and safety of UST in vulnerable populations (e.g., children, pregnant women, and the elderly) still lack real-world data. Most of the data on the treatment with UST is mainly from the United States and Europe, and there is an urgent need for data from diverse populations. Besides, data on the benefits and risks of the combination of UST with other therapeutic candidates, requires attention. Considerable efforts are needed to identify prognostic and predictive biomarkers to aid early recognition, diagnosis, stratification, treatment, and monitoring, which help doctors implement the TDM and deliver precision medicine in IBD. Despite the high safety profile of UST, more emphasis should be placed on the occurrence of specific safety events, such as skin reactions and infections, and the research in this area is still insufficient.

This bibliometric study systematically analyzed the basic situation, research hotspots, and developmental trend of ustekinumab in the clinical treatment of Crohn’s disease from a visual perspective, offering comprehensive guidance for clinicians and scholars engaged in this field. With its great efficacy, and high safety in the treatment of CD, UST has the potential to be an alternative new therapeutic candidate after the failure of conventional immunosuppressive therapy or TNF-α antagonists. Undoubtedly, the next few years will see a spectacular increase in clinical studies, supplementing real-world data on ustekinumab therapy for the precise treatment and management of CD.

### 4.4 Limitation

Although CiteSpace was used for bibliometric study, some limits were unavoidably created. Even though one of the most thorough, systematic, and reliable databases for data acquisition is the WoSCC database, which is frequently used for bibliometric analysis and visualization of scientific literature, doing so alone and excluding data from other databases may have led to bias. Additionally, the strict retrieval strategy might have led to some data loss. Also, only full articles were included in this study, and the credibility of the results largely depended on the quality of the analyzed literature. Nevertheless, most of the articles regarding ustekinumab and Crohn’s disease were included in this study as far as possible, and the visual analysis based on the aforementioned articles offered scholars the opportunity to grasp the actual research hotspots, the evolution of research, and progressing trends in this field.

## 5 Conclusion

Ustekinumab possesses important research value and is a cutting-edge drug candidate in the clinical application of Crohn’s disease, especially for adult patients with moderate to severe CD who have failed traditional drug treatment or have been non-responsive or intolerant to TNF-α blockers. Globally, the United States and Canada were the main leading countries, which contributed a huge share of clinical research on UST, and related research has been on the rise. Among research institutions, the Icahn School of Medicine at Mount Sinai was the one with the highest publication output. Other countries and institutions are recommended to ramp up cross-border research collaborations and exchange information. William J. Sandborn and Brian G. Feagan were prominent contributors to this field. Most of the articles on UST in the treatment of CD were cited by influential international journals, indicating that a wide range of scholars lavished much attention on UST research. Current studies focus on the efficacy, safety, therapeutic drug monitoring, indications for vulnerable populations, and biomarker discoveries, setting the stage for individualized and precision medicine for IBD. As an alternative to conventional immunosuppressive therapy or TNF-α antagonists, UST is an effective and safe therapy in real-world refractory CD patients, and its great therapeutic value enables UST to be used in the clinical treatment of CD in the future.

## Data Availability

The original contributions presented in the study are included in the article/Supplementary material, further inquiries can be directed to the corresponding authors.
